# Diffusion-weighted imaging in addition to contrast-enhanced MRI in identifying complete response in HER2-positive breast cancer

**DOI:** 10.1007/s00330-024-10857-7

**Published:** 2024-07-05

**Authors:** Anna van der Voort, Kay J. J. van der Hoogt, Ronni Wessels, Robert-Jan Schipper, Jelle Wesseling, Gabe S. Sonke, Ritse M. Mann

**Affiliations:** 1 Department of Medical Oncology, the Netherlands Cancer Institute, Amsterdam, The Netherlands; 2 Department of Radiology, the Netherlands Cancer Institute, Amsterdam, The Netherlands; 3 GROW School for Oncology and Reproduction, Maastricht University Medical Center, Maastricht, The Netherlands; 4 Department of Surgery, Catharina Hospital, Eindhoven, The Netherlands; 5 Department of Pathology, Netherlands Cancer Institute, Amsterdam, The Netherlands; 6 University of Amsterdam, Amsterdam, The Netherlands; 7 Department of Radiology and Nuclear Medicine, Radboud University Medical Center, Nijmegen, The Netherlands

**Keywords:** Diffusion-weighted imaging, Magnetic resonance imaging, HER2 positive breast cancer, Neoadjuvant therapy, Response

## Abstract

**Objectives:**

The aim of this study is to investigate the added value of diffusion-weighted imaging (DWI) to dynamic-contrast enhanced (DCE)-MRI to identify a pathological complete response (pCR) in patients with HER2-positive breast cancer and radiological complete response (rCR).

**Materials and methods:**

This is a single-center observational study of 102 patients with stage I-III HER2-positive breast cancer and real-world documented rCR on DCE-MRI. Patients were treated between 2015 and 2019. Both 1.5 T/3.0 T single-shot diffusion-weighted echo-planar sequence were used. Post neoadjuvant systemic treatment (NST) diffusion-weighted images were reviewed by two readers for visual evaluation and ADCmean. Discordant cases were resolved in a consensus meeting. pCR of the breast (ypT0/is) was used to calculate the negative predictive value (NPV). Breast pCR-percentages were tested with Fisher’s exact test. ADCmean and ∆ADCmean(%) for patients with and without pCR were compared using a Mann-Whitney U-test.

**Results:**

The NPV for DWI added to DCE is 86% compared to 87% for DCE alone in hormone receptor (HR)-/HER2-positive and 67% compared to 64% in HR-positive/HER2-positive breast cancer. Twenty-seven of 39 non-rCR DWI cases were false positives. In HR-positive/HER2-positive breast cancer the NPV for DCE MRI differs between MRI field strength (1.5 T: 50% vs. 3 T: 81% [*p* = 0.02]). ADCmean at baseline, post-NST, and ∆ADCmean were similar between patients with and without pCR.

**Conclusion:**

DWI has no clinically relevant effect on the NPV of DCE alone to identify a pCR in early HER2-positive breast cancer. The added value of DWI in HR-positive/HER2-positive breast cancer should be further investigated taken MRI field strength into account.

**Clinical relevance statement:**

The residual signal on DWI after neoadjuvant systemic therapy in cases with early HER2-positive breast cancer and no residual pathologic enhancement on DCE-MRI breast should not (yet) be considered in assessing a complete radiologic response.

**Key Points:**

- *Radiologic complete response is associated with a pathologic complete response (pCR) in HER2+ breast cancer but further improvement is warranted.*
- *No relevant increase in negative predictive value was observed when DWI was added to DCE.*
- *Residual signal on DW-images without pathologic enhancement on DCE-MRI, does not indicate a lower chance of pCR.*

## Introduction

Treatment with neoadjuvant chemotherapy plus anti-HER2-directed agents results in high pathological complete response (pCR) rates and excellent survival outcomes [1–8]. Patients who do not reach a pCR of the breast and/or lymph nodes after neoadjuvant systemic treatment (NST) face an inferior prognosis and are candidates for adjuvant treatment with trastuzumab emtansine [9]. Conversely, patients with a complete early treatment response during NST are candidates for image-guided trials to de-escalate systemic or surgical treatment [10–14]. Such de-escalation trials need a high negative predictive value (NPV) since residual invasive disease requires extended treatment.

In clinical practice, dynamic contrast-enhanced magnetic resonance imaging (DCE-MRI) is widely used for response evaluation of breast tumors during and after NST. However, the NPV of a radiological complete response (rCR) on DCE-MRI for identifying a pCR breast after NST in HER2-positive patients remains suboptimal and differs between hormone-receptor (HR)-positive (42–78%) and HR-negative breast cancer (69–88%) [15–20].

DWI is a functional MRI-technique based on the random motion of water molecules in tissue microstructures. Within oncologic imaging, DWI can be used for identifying hyper-cellular structures (i.e., tumors) by showing hindered diffusion. To quantify this, different b-value images can be used to calculate the apparent diffusion coefficient (ADC)-map. DWI can also be used to identify longitudinal microstructural changes in diffusivity due to NST. ADC difference over time (∆ADC) is reported as a potential imaging biomarker [21]. However, no externally validated thresholds for ADC values or ∆ADC percentages are currently available for post-NST-scans in HER2-positive patients specifically [22]. Also, the role of both quantitative and qualitative DWI evaluation in HER2-positive breast cancer remains unclear since most studies do not differ between breast cancer subtypes despite the difference in accuracy for identifying residual disease of DCE-MRI [23–26].

A residual signal on DW-images after NST is sometimes observed in breast cancer when DCE shows no residual enhancement. To be able to use MRI breast in image-guided systemic treatment de-escalation in patients with a pathological complete response, a high NPV is essential. Therefore, we investigated if adding DWI to standalone DCE-MRI increases the NPV in stage I-III HER2-positive breast cancer patients with no residual pathologic enhancement after NST.

## Materials and methods

### Patients

All patients with stage I-III HER2-positive breast cancer treated in the Netherlands Cancer Institute between January 2015 and March 2019 were identified. Inclusion criteria for this observational study were: (1) treatment with trastuzumab-based NST, (2) baseline and post-NST breast MRI available, (3) rCR on DCE-MRI breast after NST, and (4) breast surgery. This study was approved by the institutional review board without the need to obtain informed consent.

All included patients had confirmed HER2-positive invasive carcinoma on biopsy prior to treatment. HER2 positivity is defined as a score 3 + in immunohistochemistry, or scored as 2 + together with the amplification of the HER2-encoding gene assessed with in situ hybridization. Tumors with ≥ 10% estrogen and/or progesterone receptor expression were considered HR-positive. A breast MRI-scan was performed at baseline and after NST for response evaluation. After neoadjuvant treatment, all patients underwent breast-conserving surgery or a mastectomy and axillary staging with sentinel lymph node biopsy, MARI procedure [27, 28], or axillary lymph node dissection.

### MRI acquisition

Patients were scanned in a prone position using a 1.5-T or 3.0-T MRI scanner (Achieva, Philips Medical Systems) with a dedicated breast array coil. The MRI protocol consisted of DCE and DWI acquisitions. DCE was based on gradient echo sequences, acquiring several 3D-T1s (T1W High-Resolution Isotropic Volume Examination (THRIVE)), starting with one pre-contrast (i.e., unenhanced) scan, followed by a series of post-contrast scans. Fifteen (15) mL gadolinium-based contrast agent (Dotarem®, Guerbet) was administered to provide extra T1 contrast. From the DCE-scan series, subtracted wash-in and wash-out images were calculated on the scanner workstation. Moreover, between the pre-contrast and first post-contrast 3D-T1, dynamic ultrafast (4D-perfusion) scans were performed for characterizing lesions’ inflow curve characteristics.

DWI preceded the DCE acquisition, using a single shot-echo planar imaging (SS-EPI) sequence using SPectral Attenuated Inversion Recovery (SPAIR) fat suppression and SENSitivity Encoding (SENSE) for parallel imaging, with b values varying from b0 to b1500. To minimize pseudo-diffusion/perfusion effects, corresponding ADC maps were calculated based on b = 150 s/mm^2^ and b = 800 s/mm^2^, if available [29]. Sequence parameters are reported in the Supplementary Material (Supplementary Material, page 2).

### Image analysis

Baseline and post-NST DCE-MRI scans were evaluated as part of routine clinical practice. rCR as observed on the DCE scans was retrieved from the radiology reports as previously described by Van Ramshorst et al [15]. After reviewing all reports, ambiguous cases were revised by a dedicated breast radiologists (R.W., reader 1). The definition used for rCR on DCE-MRI was the absence of pathological contrast enhancement in the original tumor region. Therefore, minimal remaining contrast enhancement in the original tumor region similar to or less than surrounding normal breast tissue was considered physiological. All cases with rCR on DCE-MRI were subsequently assessed by two dedicated readers. Reader 1 had 4 years of experience in reading MRI breast and reader 2 had 16 years of experience in MRI breast. A standardized reading form was used, endorsed by both readers who evaluated the available images using the picture archiving and communication system.

**Fig. 1:**
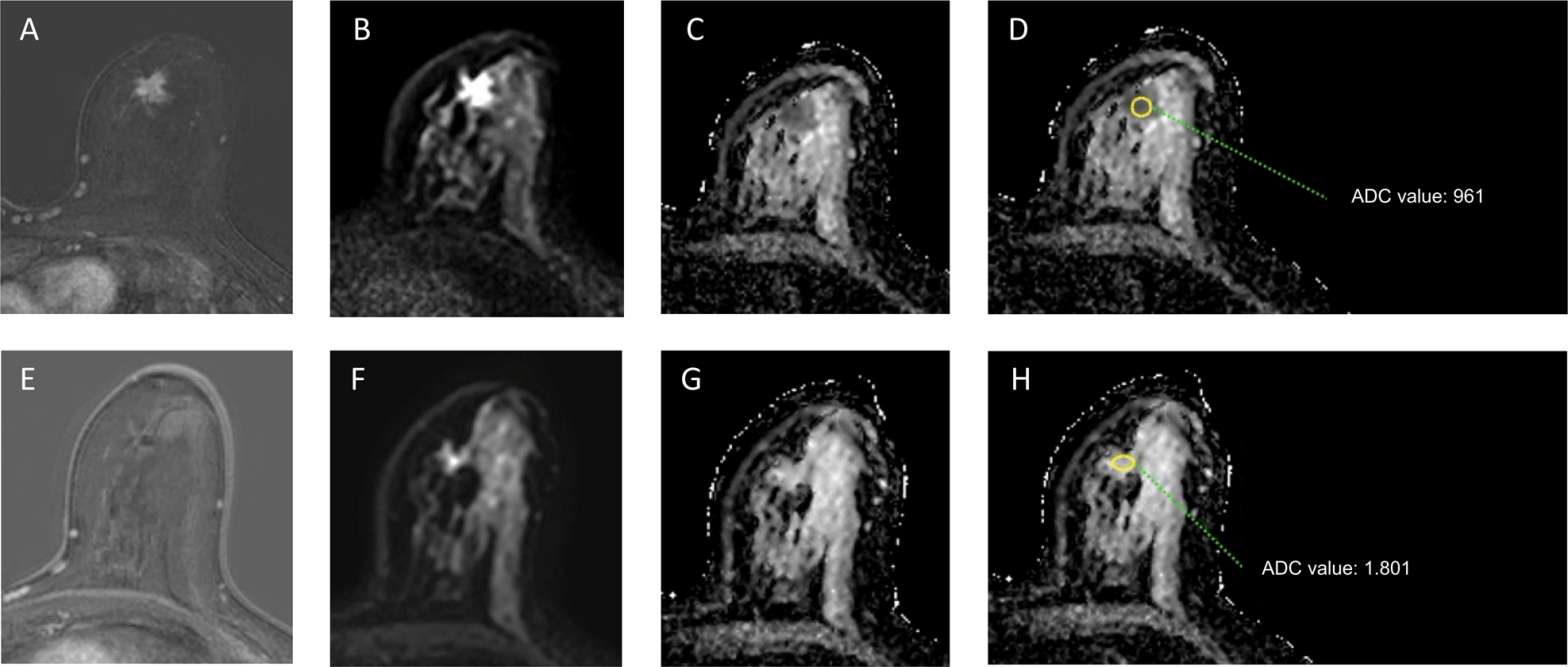
Assessment of ADC value in a patient with radiological complete response at contrast-enhanced MRI breast. **A–D** show pre-NST images and **E**–**H** show the post-NST images. **A**, **E** are subtracted images showing the tumor area (the dark spot in **E** is due to the clip inserted at the tumor site). **B**, **F** show the corresponding area at the b800 images. **C**, **G** show the corresponding ADC maps. **D**, **H** show the ROIs used with the corresponding ADC values in 10^−6^ mm^2^/s

The reading protocol consisted of the following items on the pre-treatment scans: quality of DWI-MRI (good/moderate/bad), region of interest (ROI), and ADCmean [mm^2^/s] (Supplementary Material, page 4). The quality of the DW-images was good when the image was reasonable free of distortion, ghosting, and chemical shift artifacts, and had adequate fat suppression. When there were evident artifacts, but interpretation could be performed, the image quality was labeled moderate. The readers were instructed to select an oval ROI on the ADC-map, corresponding to the most pronounced appearing hindered diffusion region on the high DW-image (Fig. 1). In case lesions covered multiple slices, the middle cross-sectional area on the ADC-map was used. Subsequently, also post-NST DWI-MRI scan quality was scored, followed by the items: high signal at b0 (yes/no), high signal at high DW-image ( ≥ b800) (yes/no), tumor visible at ADC-map (yes/no), characteristics, DWI-MRI rCR conclusion (Supplementary Material, page 4). The ROI for the ADC values on post-NST scans was placed within the former tumor region that still showed a relative high signal on high b-value images (≥ b800, using the baseline MRI as a reference). If the tumor was no longer visible, a landmark (marker or anatomic structure) could be used to support post-NST identification on DWI. The final conclusion for the DWI-MRI rCR reading was scored as follows: definitely rCR, probably rCR, possible rCR, or definitely no rCR. Disagreements on the final DWI-MRI conclusion were resolved in a consensus meeting. Only cases scored with definitely rCR by both readers after the consensus meeting were considered rCR on DCE and DWI.

## Endpoints

The primary endpoint for this study was pCR defined as the absence of residual invasive cancer (ypT0/is) on microscopic evaluation of the resected breast specimen. After surgery, all resection specimens were evaluated for any residual invasive disease by a dedicated breast cancer pathologist according to guideline-driven routine clinical practice. In case of doubt, immunohistochemistry for keratins (CAM 5.2) was performed to check whether remaining epithelial cancer cells were detected or not. As a secondary endpoint, we evaluated pCR breast defined as the absence of residual invasive cancer (ypT0). The NPV for DCE with and without DWI was calculated as the percentage of true negative patients (rCR plus pCR) within all patients with rCR. The change in ADCmean after NST was calculated as the percentage difference between post-NST and baseline ADCmean $\left(\frac{{ADCmean}\,{{{{\rm{post}}}}}\mbox{-}{{{{\rm{NST}}}}}{-}{ADC}{{{{\rm{mean}}}}} \, {{{\rm{pre}}}}\mbox{-}{{{\rm{NST}}}}}{{ADCmean}\,{{{\rm{pre}}}}\mbox{-}{{{\rm{NST}}}}}\times 100 \%\right)$. For ADCmean analysis only values within the range 0.50 × 10^−3^ mm^2^/s–3.0 × 10^−3^ mm^2^/s were included in the analysis to minimize the effects of unrealistic outliers.

### Statistical analysis

All statistical analyses were performed using SPSS version 27.0. Differences in clinical and treatment characteristics between patients with and without pCR were analyzed using an independent t-test for continuous and normally distributed data, a Mann-Whitney U test for continuous and not normally distributed data, and a Fisher’s exact test for the categorical variables. Pre- and post-NST ADCmean distributions and ∆ADCmean (%) were compared for pCR/non-pCR using a Mann-Whitney U test. A Cohen’s kappa (κ) was used to test inter-reader variability. For all statistical analyses a *p*-value < 0.05 was considered statistically significant.

## Results

### Patients

**Fig. 2:**
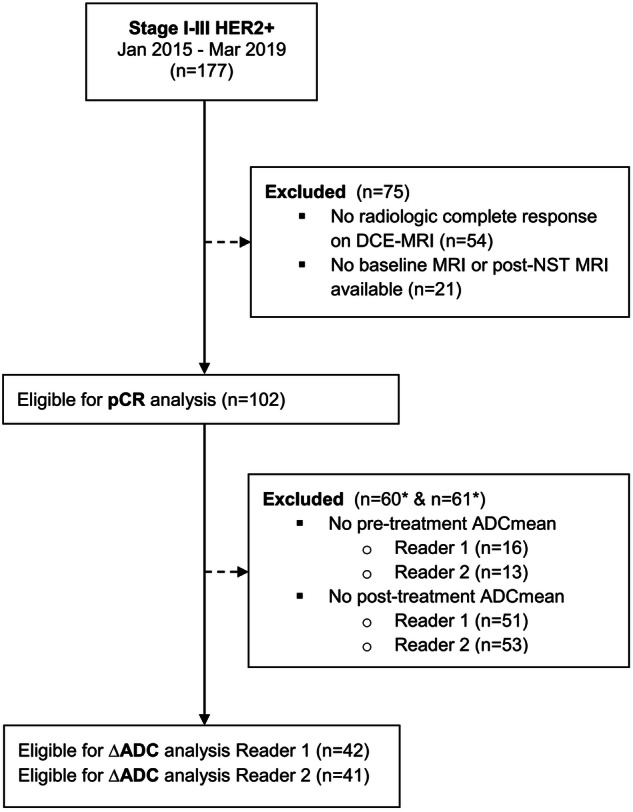
Study flow chart. Abbreviations: DCE, dynamic contrast-enhanced; pCR, pathological complete response; NST, neoadjuvant systemic therapy; ADC, apparent diffusion coefficient. *Numbers do not add up because in 7 cases for Reader 1, both pre-treatment ADCmean and post-treatment ADCmean were not available, and in 5 cases for Reader 2, both pre-treatment and post-treatment ADC mean were not available

We identified 177 patients with stage I-III HER2-positive breast cancer treated from January 2015 until March 2019 (Fig. 2). Of these, 123 had no residual pathological enhancement on post-NST DCE-MRI and were therefore considered as radiologic complete responders. Of them, 102 had MRI scans available at baseline and post-NST.

Most patients (*N* = 71) received a combination of paclitaxel, carboplatin, trastuzumab and pertuzumab, as standard-of-care treatment [5]. Table 1 shows the patient and tumor characteristics.

**Table 1:** Patient and tumor characteristics by patients with pathological complete response of the breast or residual invasive disease after neoadjuvant chemotherapy

	All patients	pCR breast (ypT0/is)	Non-pCR	
	*N* = 102	*N* = 76	*N* = 26	*p*-value
Age, mean ± SD (years)	46.9 ± 11.9	46.2 ± 10.5	48.9 ± 12.0	0.28
Baseline size, mean (mm)	37.7	39.3	33.0	0.98
**Clinical stage**				0.49
I	13 (12.7%)	9 (11.8%)	4 (15.4%)	
II	60 (58.8%)	43 (56.6%)	17 (65.4%)	
III	29 (28.4%)	24 (31.6%)	5 (19.2%)	
**Clinical lymph node stage**				0.49
cN0	48 (47.1%)	39 (51.3%)	9 (34.6%)	
cN1	35 (34.2%)	23 (30.3%)	12 (46.2%)	
cN2	7 (6.9%)	5 (6.6%)	2 (7.7%)	
cN3	12 (11.8%)	9 (11.8%)	3 (11.5%)	
**Tumor grade**				0.61
2	40 (39.2%)	32 (42.1%)	8 (30.8%)	
3	58 (56.9%)	41 (53.9%)	17 (65.4%)	
Unknown	4 (3.9%)	3 (3.9%)	1 (3.9%)	
**Histology**				0.47
No special type	91 (89.2%)	69 (90.8%)	22 (84.6%)	
Other	11 (10.8%)	7 (9.2%)	4 (15.4%)	
**Location**				0.77
Upper outer quadrant	31 (30.4%)	21 (27.6%)	10 (38.5%)	
Overlapping quadrants	34 (33.3%)	25 (32.9%)	9 (34.6%)	
Central	14 (13.7%)	12 (15.8%)	2 (7.7%)	
Other quadrants	22 (21.6%)	17 (22.4%)	5 (19.2%)	
Bilateral breast cancer	1 (1.0%)	1 (1.3%)	0 (0.0%)	
**Enhancement at baseline**				0.89
Mass-enhancement	70 (68.6%)	51 (67.1%)	19 (73.1%)	
Non-mass enhancement	14 (13.7%)	14 (18.4%)	4 (15.4%)	
Combined	18 (17.6%)	11 (14.5%)	3 (11.5%)	
**Menopausal status**				0.60
Premenopausal	61 (59.8%)	47 (61.8%)	14 (53.8%)	
Perimenopause	11 (10.8%)	7 (9.2%)	4 (15.4%)	
Postmenopausal	27 (26.5%)	19 (25.0%)	8 (30.8%)	
Unknown	3 (2.9%)	3 (3.9%)	0 (0.0%)	
Hormone receptor-status*				0.01
Positive	56 (54.9%)	36 (47.4%)	20 (76.9%)	
Negative	46 (45.1%)	40 (52.6%)	6 (23.1%)	
**NST regimen**				0.97
PTC-Ptz*	71 (69.6%)	53 (69.7%)	18 (69.2%)	
AC/FEC & PT±Ptz	24 (23.5%)	18 (23.7%)	6 (23.1%)	
PT	7 (6.9%)	5 (6.6%)	2 (7.7%)	
**MRI-field strength post NST**				0.02
1.5 T	52 (51.0%)	34 (44.7%)	18 (69.2%)	
3 T	49 (48.0%)	42 (55.3%)	7 (26.9%)	
Unknown	1 (1.0%)	0 (0.0%)	1 (3.8%)	
**Surgery**				0.60
Mastectomy	25 (24.5%)	20 (26.3%)	5 (19.2%)	
BCS	77 (75.5%)	56 (73.7%)	21 (80.8%)	

*SD* standard deviation, *HR* hormone receptor, *pCR* pathological complete response, *NST* neoadjuvant systemic therapy, *PTC-Ptz* Paclitaxel, trastuzumab, carboplatin and pertuzumab, *AC* adriamycine and cyclophosphamide, *FEC 5-Fluorouracil* epirubicine and cyclophosphamide, *PT ± Ptz* Paclitaxel, trastuzumab with or without pertuzumab, *BCS* breast-conserving surgery. * One patient was treated with trastuzumab, carboplatin, and docetaxel (TCH) * Tumors were considered hormone receptor-positive if more than 10% of the cells were positive for either the estrogen receptor or progesterone receptor

Three-quarters of all included patients (76%) underwent breast-conserving surgery, and 24% mastectomy as definite surgery. The median time between the baseline MRI and the post-NST MRI was 196 days (interquartile range [IQR] 177–208 days), and 27 days (IQR 19–33 days) between post-NST MRI and surgery. After NST 76 patients had a confirmed pCR and 26 had not, resulting in a NPV for DCE-MRI of 75%. Residual invasive tumor in the resection specimen was mostly smaller than 2 cm (ypT1: 77%). HR-positive breast cancer was associated with a reduced likelihood of pCR between HR-negative and positive cohorts (87% vs. 64%, *p* = 0.01, Table 1). Also, patients with rCR on a 3-T DCE-MRI scan had higher chance of pCR than patients with rCR on 1.5 T MRI scan (86 vs 65%, *p* = 0.02), more pronounced within the HR-positive subgroup (50 vs. 81%, *p* = 0.03) than in HR-negatives (88% vs. 91%). Therefore, we present the qualitative analyses separately for the two HR-subgroups and for MRI field strength.

### Imaging characteristics

The majority of the post-NST DWI-MRI scans was of good quality (Supplementary Material, page 3). Reader 1 scored 98 out of 102 cases (96%) as good quality, and reader 2 89 of 102 (87%). The level of inter-reader variability on the scored elements was high, with a poor to slight agreement score except for visibility at the b0-image (κ = 0.38) and tumor characteristics (κ = 0.20) on which the level was fair. Reader 2 scored more non-pCR cases than pCR cases as visible on b0-images. A hyper-intense residual signal at high b-value images did not differentiate between pCR and non-pCR cases as scored by both readers. For most patients the readers used (> 70%) b800 as the high b-value for determining residual signal at the original tumor location.

### Qualitative DWI evaluation

**Fig. 3:**
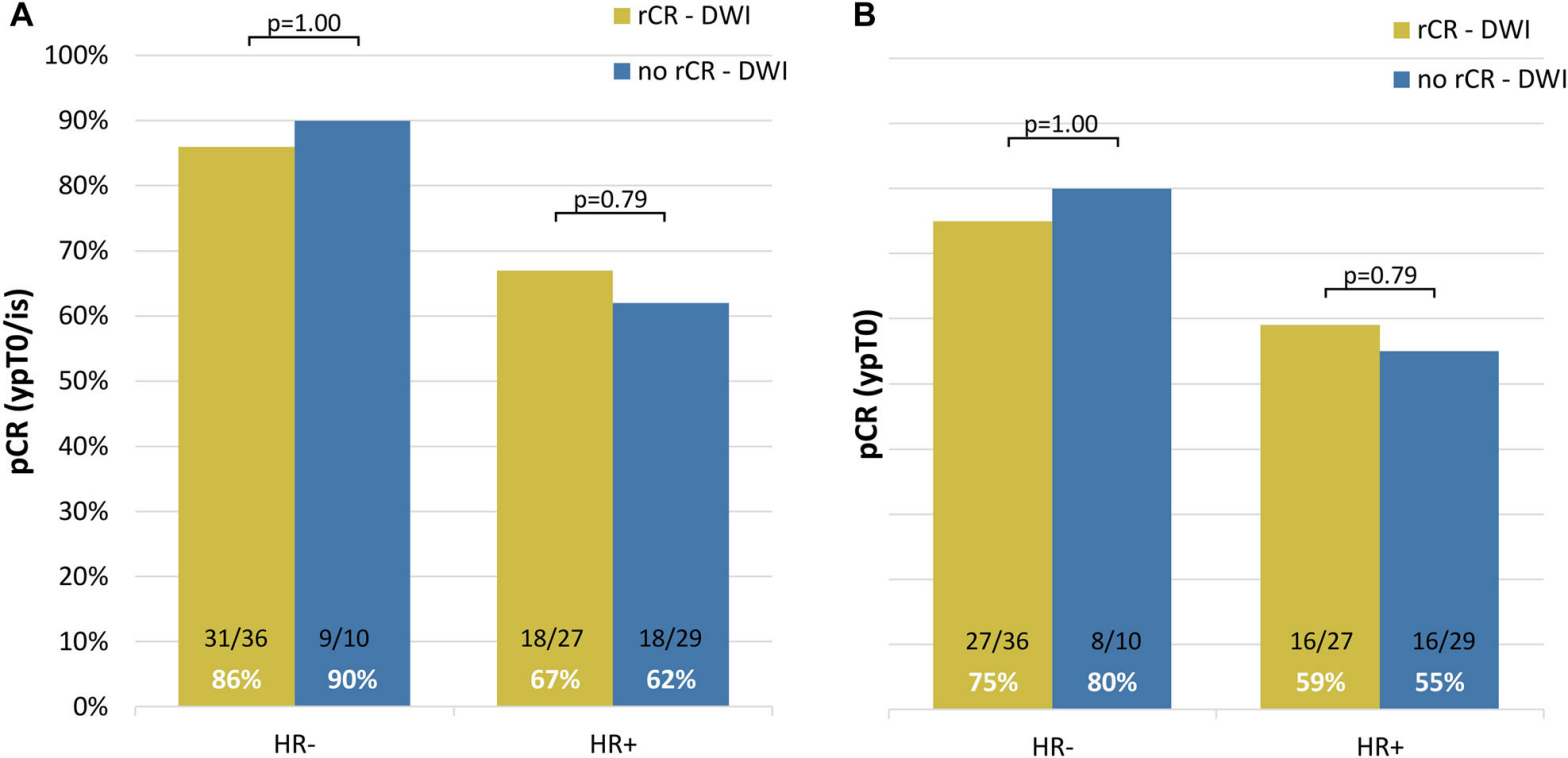
Pathological complete response (pCR) percentages per hormone receptor (HR)-subgroup in patients with and without a radiologic complete response (rCR) based on qualitative DWI evaluation. **A** results for a pCR defined as the absence of invasive residual disease (ypT0/is). **B** results for pCR defined as the absence of residual invasive and in situ disease (ypT0). pCR, pathological complete response; HR, hormone receptor; DWI, diffusion-weighted imaging

We found a definite rCR on combined DCE- and DWI-MRI in 63 patients. The corresponding overall NPV for combined DCE-DWI is 78% (Table 2). Twenty-seven of 39 non-rCR DWI cases (69%) were false positives. Twelve of 39 non-rCR DWI cases had residual invasive disease (31%) and were true positives. Within the HER2-positive/HR-negative subgroup the NPV is 87% for DCE-only and 86% for combined DCE-DWI. In HER2+ /HR+ breast cancer patients the NPV for DCE alone is 64% and combined with DWI the NPV is 67%. Results for subgroups based on the MRI field strength are also shown in Table 2. The NPV at 1.5 T increases from 50% with DCE-only to 56% when DWI is added to the response evaluation, but remains lower than 3-T MRI evaluation for both DCE and DCE plus DWI. The pCR-percentages for rCR and non-rCR cases on DWI-MRI for both pCR breast definitions (ypT0/is, *N* = 76 and ypT0, *N* = 71) are presented in Fig. 3. Results for the alternative pCR definition without in situ disease (ypT0) show great overlap with the primary endpoint, only three cases of the 27 false positive DWI cases had residual ductal or lobular carcinoma in situ.

**Table 2:** Pathological complete response (pCR) percentages for patients with rCR on DCE or DCE combined with DWI and corresponding negative predictive value (NPV) per hormone receptor-subgroup and per magnetic field strength

MRI	Post NST field strength	Subtype	pCR (*N* = 76)	Non-pCR (*N* = 26)	Total (*N* = 102)	NPV*
**rCR DCE**	1.5 T/3 T	HER2+	76	26	102	75%
		HER2+/HR-	40	6	46	87%
		HER2+/HR+	36	20	56	64%
**rCR DCE** + **DWI**	1.5 T/3 T	HER2+	49	14	63	78%
		HER2+/HR-	31	5	36	86%
		HER2+/HR+	18	9	27	67%
**rCR DCE**	1.5 T	HER2+	34	18	52	65%
		HER2+/HR-	19	3	22	86%
		HER2+/HR+	15	15	30	50%
**rCR DCE** + **DWI**	1.5 T	HER2+	24	9	33	73%
		HER2+/HR-	15	2	17	88%
		HER2+/HR+	9	7	16	56%
**rCR DCE**	3 T	HER2+	42	7	49	86%
		HER2+/HR-	21	2	23	91%
		HER2+/HR+	21	5	26	81%
**rCR DCE** + **DWI**	3 T	HER2+	25	4	29	86%
		HER2+/HR-	16	2	18	89%
		HER2+/HR+	9	2	11	82%

*DWI* diffusion-weighted imaging, *DCE* dynamic contrast-enhanced, *pCR* pathological complete response, *rCR* radiological complete response, *HR*- hormone receptor-negative, *HR+*  hormone receptor-positive, *rCR DCE* *+* *DWI-MRI* no residual pathological enhancement on DCE-MRI and scored as ‘definitely rCR’ at DWI in consensus by reader 1&2

### Quantitative DWI evaluation

**Fig. 4:**
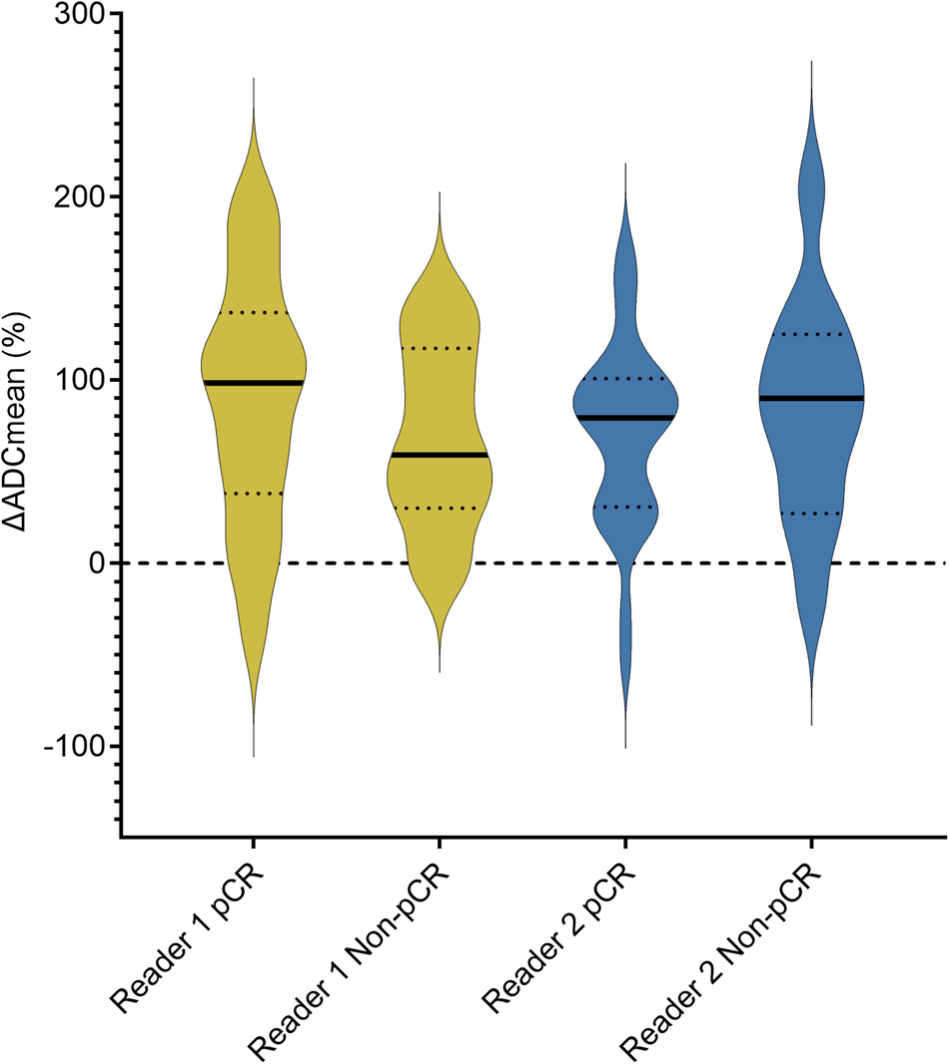
Violin plot ∆ADCmean (%), from baseline to post-NST, per reader in patients with and without a pathological complete response (pCR). ADC, apparent diffusion coefficient; pCR, pathological complete response

Baseline ADCmean could not be determined in nine cases by reader 1 due to technical issues and seven were non-realistic outliers and therefore excluded. Baseline ADCmean could not be determined in 13 cases by reader 2 due to technical issues. Post-NST ADCmean could be determined in patients in which the readers could identify the original tumor location and were able to place a ROI (Fig. 2). If the former tumor location could not be determined due to no residual signal on DWI and no valid landmark the readers were not able to draw a ROI. Furthermore, 2 cases had non-realistic outlier ADC values and were excluded in the quantitative analysis. From the included 102 patients a ∆ADC(%) could be calculated in 42 cases by reader 1 and in 41 cases by reader 2. Baseline ADCmean were 0.84 ± 0.21 × 10^−3^ mm^2^/s and 0.89 ± 0.33 × 10^−3^ mm^2^/s in patients with and without pCR for reader 1 (*p* = 0.88) and respectively 0.95 ± 0.24 × 10^−3^ mm^2^/s and 0.89 ± 0.28 × 10^−3^ mm^2^/s (*p* = 0.15) for reader 2 (Table 3). Post-NST ADCmean were respectively 1.55 ± 0.51 × 10^−3^ mm^2^/s versus 1.37 ± 0.44 × 10^−3^ mm^2^/s (*p* = 0.22) by reader 1 and 1.62 ± 0.43 × 10^−3^ mm^2^/s versus 1.55 ± 0.39 × 10^−3^ mm^2^/s (*p* = 0.56) by reader 2. Overall, most patients showed an increase in ADCmean after NST, reflected in a positive ∆ADCmean percentage. However, no statistically significant differences were found between patients with or without pCR. DeltaADCmean percentages are also shown by MRI field strength and show no significant differences between pCR and non-pCR cases, but numbers are low since only 48 patients were scanned using the same field strength pre-and post-NST. The variance between the measurements for pCR/non-pCR, quantified by the percentage change in ADCmean after NST, is demonstrated in Fig. 4.

**Table 3:** Differences in ADCmean between patients per reader according to pathological complete response (pCR) of the breast (ypT0/is)

		Reader 1				Reader 2		
		pCR	Non-pCR			pCR	Non-pCR	
ADC mean × 10^−3^ mm^2^/s	N	mean ± SD	mean ± SD	*p*-value	N	mean ± SD	mean ± SD	*p*-value
**HR-/HER2**+								
Baseline ADCmean	40	0.86 ± 0.16	0.86 ± 0.22	0.82	42	0.99 ± 0.23	0.95 ± 0.23	0.59
Post NST ADCmean	26	1.50 ± 0.51	1.32 ± 0.53^a^	0.39	19	1.49 ± 0.51	1.69 ± 0.21^a^	0.71
∆ADCmean in %	23	77% ± 58%	35% ± 64%^a^	0.52	17	56% ± 62%	62% ± 32%^a^	0.95
**HR**+ **/HER2**+								
Baseline ADCmean	46	0.83 ± 0.26	0.90 ± 0.38^a^	0.51	47	0.90 ± 0.24	0.87 ± 0.30	0.37
Post NST ADCmean	25	1.65 ± 0.50	1.38 ± 0.44^a^	0.36	30	1.73 ± 0.31	1.51 ± 0.43	0.23
∆ADCmean in %	19	86% ± 16%	86% ± 22%	0.66	24	84% ± 35%	92% ± 69%	0.57
**Total HER2**+								
Baseline ADCmean	86	0.84 ± 0.21	0.89 ± 0.33	0.88	89	0.95 ± 0.24	0.89 ± 0.28	0.11
Post NST ADCmean	51	1.55 ± 0.51	1.37 ± 0.44	0.22	49	1.62 ± 0.43	1.55 ± 0.39	0.56
∆ADCmean in %	42	89% ± 67%	69% ± 50%	0.33	41	71% ± 51%	84% ± 61%	0.57
**Total HER2**+**,1.5** **T**								
∆ADCmean in %	7	-^a^	64% ± 54%	-	6	100%^a^	75% ± 68%^a^	-
**Total HER2**+**, 3** **T**								
∆ADCmean in %	15	109% ± 72%	61% ± 56%	0.30	8	71% ± 38%	137% ± 95%^a^	0.64

*ADC* apparent diffusion coefficient, *pCR* pathological complete response, *SD* standard deviation, *HER2+*  HER2-positive, *HR-* hormone receptor-negative, *HR+*  hormone receptor-positive, *NST* neoadjuvant systemic therapy

^a^ *n* ≤ 5

-differences in number of cases between readers show the difference in the ability to draw a ROI on the baseline and/or post-NST scan

## Discussion

We investigated the added value of qualitative and quantitative DWI-MRI in patients with stage I-III HER2-positive breast cancer with a complete radiologic remission on DCE-MRI for identifying a pCR of the breast after NST. We found that 1.5-T DCE-MRI has a lower chance of detecting residual disease than 3-T DCE-MRI in HR-positive/HER2-positive disease. We did not find a clinically relevant increase in NPV after adding qualitative DWI assessment to the standard DCE evaluation. While 39 of 102 cases had a residual signal on DWI, 27 of these were false positives and showed no residual invasive disease on pathology examination. Furthermore, we did not find a difference between pCR and non-pCR patients in terms of baseline ADCmean, post-NST ADCmean or ∆ADCmean percentage, and we observed a high inter-reader variability.

DWI in standard breast cancer evaluation protocols is generally recommended to increase the specificity of DCE-MRI alone [30]. This multi-parametric MRI approach provides the opportunity to combine vascularity and cellularity-related measurements, which helps to distinguish benign breast lesions from malignant tissue and may prevent unnecessary biopsies. We hypothesized that DWI would complement DCE to detect residual invasive disease after NST, especially in HR-positive tumors. As HR-positive tumors are less likely to reach pCR [1–5], and because the NPV of DCE-MRI post-NST in HR-positive/HER2-positive breast cancers is only around 60% [15, 17]. Also, the tumor micro-environments (TMEs) and types of responses differ between HR-subtypes with for example higher percentage of immune cells in more HER2-driven tumors. Therefore, also cellularity could differ with corresponding varying ADCs between HER2-subgroups at response evaluation [31–33]. However, we found no clinically relevant increase in NPV for HR-positive breast cancer patients by adding DWI. In addition a NPV of 67% remains not adequate. This prompts us with the question how we can further improve response evaluation for this specific subgroup. For this subgroup, we observed, despite a small subgroup size, a large difference in NPVs between 1.5 T and 3.0 T. It might be, therefore, desirable to further investigate the influence of different field strengths on pCR prediction in specifically HR-positive/HER2-positive breast cancer, in DCE as well as in DWI.

Our research does not stand on its own. A recent meta-analysis showed a high area under the curve of 0.82 for pCR detection with DWI after NST, but the performance of the combination of DWI and DCE was not investigated [24]. Hahn et al observed an increase in accuracy, specificity and positive predictive value when DWI was added to DCE in response evaluation. However, in line with our results, no increase in NPV was observed [23]. This study did not stratify their results based on breast cancer subgroups, MRI field strength or provided data on inter-reader variability. Our findings show a difference in NPV between HR subgroups as well as a difference between MRI field strengths, which importance is also reported in another recent meta-analysis [34]. These results underline the essence of analyzing the effect of magnetic field strengths in breast MRI response evaluation since a higher field strength (1.5 T vs. 3.0 T) will also significantly increase the signal-to-noise ratio, thereby not neglecting other challenges when using a higher field strength [35].

Besides visual evaluation, ADC values hold a promise of high reproducibility if measured correctly [36]. However, we observed variability in the obtained results of quantitative DWI. In literature the baseline ADCmean differs among breast cancer subtypes, and does not seem to be highly predictive for pCR within HER2-positive breast cancer [21, 26, 37–39]. Our study supports this finding in patients with a rCR on DCE-MRI after NST. Using manually assessed ADCmean alone before the start of NST, as a purely predictive biomarker, is therefore unsupported in stage I-III HER2-positive breast cancer. Nevertheless, the use of longitudinal ADCmean changes seems more promising in identifying pCR. Liu et al found higher post-NST ADCmean in pCR patients compared to patients with residual disease with a corresponding high NPV of ranging from 87% to 95% within subgroups with HER2-positive patients [38]. Two differences in study design might be attributing to the difference with our study. First, the readers in other studies were instructed to draw a ROI in visual normal breast tissue if the tumor was not visible anymore [21, 26, 38]. Our readers were instructed to draw a ROI if they could determine the original tumor area. This was mostly limited to patients who had residual high signal on b800-b1500-images resulting in less than 40% of patients in which a ADCmean difference could be calculated. In practice, we encountered that determining the ROI-location for rCR on DWI can be very challenging, especially in multi-focal disease and in patients with excellent response (complete rCR on DCE-MRI) due to distortion in the DWI scan, as inclusion of healthy unaffected fibro-glandular tissue needs to be avoided. Secondly, our analysis was performed within a highly selected group of patients with already a rCR on DCE. This implies that the post-NST-ADCmean or ADCmean difference could potentially differentiate complete responders from non-complete responders independently, but the added value to rCR on DCE-MRI seems limited.

The I-SPY and ACRIN consortiums conducted multi-center multi-scanner studies investigating the role of DWI in response prediction. Partridge et al showed that ∆ADC-percentage was predictive for pCR at early treatment scans and post-systemic treatment scans [21]. This difference was not specifically found in the HER2-positive subgroups, but only 67 HER2-positive patients were included in this analysis. Therefore, a small difference could possibly not be detected. Li et al reported functional tumor volume (FTV), and longest diameter to be the most predictive DCE features [40]. The same author group investigated the predictive value of ∆ADC combined with the quantitative DCE-feature percentage change in functional tumor volume (∆FTV%) and found a small but significant added value of ∆ADCmean(%) within HER2-positive and HR-positive patients but also did not take inter-reader variance nor MRI field strength into account [22].

## Limitations

Our study has some limitations. First, this was a retrospective study where the MRI protocols were not identical for all patients, including variability in the magnitude and b-values used. Furthermore, we selected patients with rCR on DCE-MRI. Therefore, we cannot report on accuracy, specificity or sensitivity for all women with stage I-III HER2-positive breast cancer. Second, the inter-reader variability was high for almost all scored imaging characteristics, despite organizing a pre-reading meeting in which both readers provided consent for the reading -protocol. However, this does not affect the outcomes for the qualitative analysis since a consensus reading was performed. Furthermore, the ROI-selection technique could possibly be optimized. Since manually selecting ROIs with low diffusivity showed excellent agreement and sensitivity in early response evaluation, we used a practical approach with a manually 2D-ROI selection method [41]. For future studies, (semi)automatic or 3D whole-tumor ROI selection methods could potentially improve the reproducibility of ADCs [42, 43].

## Conclusions

We did not find evidence that DWI-MRI provides a clinically relevant increase in the NPV of DCE-MRI to identify pathologic complete responders in stage I-III HER2-positive breast cancer after NST. Further studies using (semi-)automated delineations of (3D-)ROIs and including non-radiologic complete responders on DCE-MRI should be conducted to further assess the role of DWI in response evaluation after NST in HER2-positive breast cancer. Hormone-receptor subgroups and MRI field strength should always be taken into account in those future studies.

## Supplementary information

Supplementary Material

## Abbreviations

ADC: Apparent diffusion coefficient
DCE: Dynamic contrast-enhanced
DWI: Diffusion-weighted imaging
HER2: Human epidermal growth factor receptor 2
HR: Hormone receptor
MRI: Magnetic resonance imaging
NPV: Negative predictive value
NST: Neoadjuvant systemic treatment
pCR: Pathological complete response
rCR: Radiological complete response
ROI: Region of interest
SD: Standard deviation
