# Correction: Diffusion-weighted imaging in addition to contrast-enhanced MRI in identifying complete response in HER2-positive breast cancer

**DOI:** 10.1007/s00330-024-11008-8

**Published:** 2024-08-13

**Authors:** Anna van der Voort, Kay J. J. van der Hoogt, Ronni Wessels, Robert-Jan Schipper, Jelle Wesseling, Gabe S. Sonke, Ritse M. Mann

**Affiliations:** 1 Department of Medical Oncology, the Netherlands Cancer Institute, Amsterdam, The Netherlands; 2 Department of Radiology, the Netherlands Cancer Institute, Amsterdam, The Netherlands; 3 GROW School for Oncology and Reproduction, Maastricht University Medical Center, Maastricht, The Netherlands; 4 Department of Surgery, Catharina Hospital, Eindhoven, The Netherlands; 5 Department of Pathology, Netherlands Cancer Institute, Amsterdam, The Netherlands; 6 University of Amsterdam, Amsterdam, The Netherlands; 7 Department of Radiology and Nuclear Medicine, Radboud University Medical Center, Nijmegen, The Netherlands

**Correction: European Radiology**

10.1007/s00330-024-10857-7published online 05 July 2024

In the Abstract of this article, subsection Results, the sentence beginning with “In HR-negative/HER2-positive breast cancer…” should have been “In HR-positive/HER2-positive breast cancer…”.

The correct sentence is:

In HR-positive /HER2-positive breast cancer the NPV for DCE MRI differs between MRI field strength (1.5 T: 50% vs. 3 T: 81% (*p* = 0.02)).

Furthermore, in the online version of the article, In Section ‘Endpoints’ the equation for ADCmean is displayed incorrectly due to an error in copyediting.

Instead of:

$$\left(\frac{ADCmean\,{\rm{post}}{\mbox{-}}{\rm{NST}}\backslash {\rm{mbox}}\{{\mbox{-}}\}ADC{\rm{mean}}\,{\rm{pre}}{\mbox{-}}{\rm{NST}}}{ADCmean\,{\rm{pre}}{\mbox{-}}{\rm{NST}}}\times 100 \% \right)$$

The correct equation is as follows:

$$\left(\frac{ADCmean\,{\rm{post}}{\mbox{-}}{\rm{NST}}{\mbox{-}}ADC{\rm{mean}}\,{\rm{pre}}{\mbox{-}}{\rm{NST}}}{ADCmean\,{\rm{pre}}{\mbox{-}}{\rm{NST}}}\times 100 \% \right).$$

The original article has been corrected.

