# Supplementary material for: Diffusion-weighted imaging in addition to contrast-enhanced MRI in identifying complete response in HER2-positive breast cancer
Source: Eur Radiol. 2024 Jul 5;34(12):7994–8004. doi: 10.1007/s00330-024-10857-7 (PMC11557627; doi:10.1007/s00330-024-10857-7)
Supplement: Supplementary file 1 — Supplementary Material [file 330_2024_10857_MOESM1_ESM.pdf]

Diffusion-weighted imaging in addition to contrast-enhanced MRI in identifying complete response in HER2-positive breast cancer

Electronic Supplementary Material (ESM)

Content

Supp.Table 1: MRI sequence parameters p.2
Supp.Table 2: DWI-MRI reading characteristics p.3
Reading protocol and form p.4

Supp.Table 1: MRI parameters

	3D-T1/THRIVE		DWI	
	**1.5 T**	**3.0 T**	**1.5 T**	**3.0 T**
**Echo time (ms)**	1.7-2.0	1.6-2.0	86-88	57-79
**Repetition time (ms)**	3.9-6.1	3.2-3.7	3313-6909	5521-11742
**Flip angle (°)**	10	10	90	90
**Acquired Matrix (freq.×phase)**	344-440×278-416	288-440×288-416	144-160×161-177	160-192×157-192
**Reconstruction matrix (freq.×phase)**	448-640×448-640	448-525×448-528	288-480×288-480	288-448×288-448
**Echo train length**	139-143	110-144	47-51	33-53
**Pixel spacing (mm)**	0.6-0.9×0.6-0.9	0.7-0.9×0.7-0.9	0.8-1.25×0.8-1.25	0.9-1.25×0.9-1.25
**Slice thickness (mm)**	1.8-2.3	1.8	5	3-5
**Spacing between slices (mm)[ƚ]**	0.9-1.15	0.9	5.5	3.5-5.5
**Number of dynamics**	6	6	-	-
**Fat suppression**	SPAIR	SPAIR	SPAIR	SPAIR
**Coil (number of channels)**	7	7, 16	7	7, 16

*Small differences in MRI protocols are not completely ruled out. Number of averages=2 for all DWI-protocols. [ƚ]Space between slices from center-to-center. SPAIR: SPectral Attenuated Inversion Recovery; DWI= Diffusion-weighted imaging; THRIVE= T1W High Resolution Isotropic Volume Examination

Supp.Table 2. DWI-reading characteristics

	Reader 1			Reader 2			
	**pCR**	**Non-pCR**	**p-value**	**pCR**	**Non-pCR**	**p-value**	κ
**Quality baseline DWI**			0.23			0.58	0.19
Good	61 (73.1%)	19 (73.1%)		53 (69.7%)	17 (65.4%)		
Moderate	5 (6.6%)	1(3.8%)		13 (17.1%)	3 (11.5%)		
Bad	1 (1.3%)	1 (3.8%)		4 (5.3%)	3 (11.5%)		
Missing	9 (11.8%)	5 (19.2%)		6 (7.9%)	3 (11.5%)		
**Quality post DWI**			0.12			0.46	0.02
Good	74 (97.4%)	24 (92.3%)		68 (90.7%)	21 (84.0%)		
Moderate	2 (2.6%)	0 (0.0%)		7 (9.3%)	4 (16.0%)		
Bad	0 (0.0%)	2 (7.7%)		0 (0.0%)	0 (0.0%)		
Missing	0 (0.0%)	0 (0.0%)		1 (1.3%)	1 (3.8%)		
**Visible at b0**			0.33			0.04	0.38
No	55 (78%)	16 (23%)		45 (59.2%)	9 (34.6%)		
Yes	21 (68%)	10 (32%)		31 (40.8%)	17 (65.4%)		
**Visible at high b-value**			0.08			0.26	0.18
No	68 (78%)	19 (22%)		40 (52.6%)	10 (38.5%)		
Yes	7 (50%)	7 (50%)		36 (47.4%)	16 (61.5%)		
Missing	1 (1.3%)	0 (0.0%)		0 (0.0%)	0 (0.0%)		
**Visible at ADC-map**			<0.01			0.66	0.10
No	62 (81.6%)	20 (76.9%)		37 (48.7%)	10 (38.5%)		
Yes	2 (2.6%)	5 (19.2%)		33 (43.4%)	14 (53.8%)		
Unknown	12 (15.8%)	1 (3.8%)		6 (7.9%)	2 (7.7%)		
**Characteristics***			0.27			0.83	0.20
No hyper-intense signal	67 (88.2%)	20 (76.9%)		37 (48.7%)	11 (42.3%)		
Hyper-intense signal[#]	0 (0.0%)	0 (0.0%)		5 (6.6%)	1 (3.8%)		
Signal[†] & low ADC	0 (0.0%)	0 (0.0%)		5 (6.6%)	2 (7.7%)		
Signal[†] & high ADC	8 (10.5%)	6 (23.1%)		23 (30.3%)	11 (42.3%)		
Other	1 (1.3%)	0 (0.0%)		6 (7.9%)	1 (3.8%)		

Abbreviations: DWI = diffusion-weighted imaging; pCR = pathological complete response
*Characteristics at (former) tumor location; [#]small but hyper-intense signal at high b-value (b800 ≤ b-value ≤ b1500); [†] obvious hyper-intense signal at high b-value (b800≤b-value≤ b1500)

***The additive value of DWI in pCR prediction with MRI breast in HER2+ BC**: reading protocol & form*

Radiologist: ______________________________ *date (reading):* ________________________

DWI Study Number: _______________________ *Year of birth:* ________________________

1. ***DCE-MRI: breast (baseline)*** ____________ ***(dd-mm-yy)***

Remarks:

---

2. ***DW-MRI: breast (baseline)***

Quality DW-MRI: |___| *1 = Good; 2 = Moderate; 3 = Bad*

What b-value combination (in s/mm^2) used for ADC maps?

☐ *b150-b800* ☐ *b500-b1500* ☐ *other, specify* |______________|

ROI cross-sectional area [a]*:* |______| *mm^2* *Scan nr.* |______| *Slice nr.* |______|

ADC_{mean} |_______________| *(e.g. mm^2/s)*

[a] *Use region with most pronounced diffusion restriction/hindrance; Marker(if visible)/anatomic structures as a landmark; selection as big as possible, thereby excluding necrosis and fibrosis and parenchyma; If lesion covers multiple slices with pronounced diffusion hindrance/restriction use middle cross-sectional area on ADC-map.*

Remarks:

---

3. ***DCE-MRI: breast (post-NST)****:* ______________ ***(dd-mm-yy)***

Radiologic complete response on DCE-MRI: the absence of pathologic (i.e. non-physiological) contrast enhancement in the original tumor region. Remaining minimal contrast enhancement similar or less than surrounding or contralateral normal breast tissue is considered physiological.

Response breast: |___| *1 = Definitely radiologic complete response*
2 = Probably complete radiologic response
3 = Possible complete radiologic complete
4 = Probable no radiologic complete response
5 = Definitely no radiologic complete response
9 = not evaluable (if so please disclose why)

Remarks:

---

4. DW-MRI: breast (post-NST)

Quality DW-MRI: |___| 1 = Good; 2 = Moderate; 3 = Bad

High signal intensity at b0 images (primary T2-weighted)? |___| 0 = No; 1 = Yes

DWI Study Number: ______________________ Year of birth: _______________________

High signal intensity at high b-value images? |___| 0 = No; 1 = Yes

If 1 (=Yes) what b-value most informative? (≤b2000)

☐b800 ☐b1500 ☐other, specify |___________|

What b-value combination (in s/mm²) used for ADC maps?

☐b150-b800 ☐b500-b1500 ☐other, specify |_____________|

Tumor visible at ADC map: |___| 0 = No; 1 = Yes; 2 = NA (if not available)

ROI cross-sectional area [b]:|______| mm² Scan nr. |______| Slice nr. |______|

ADC_{mean}|_______________| (e.g. mm²/s)

[b] Use region with most pronounced diffusion restriction/hindrance with marker/anatomic structures as a landmark; selection as big as possible (excl. fibrosis, necrosis etc.)/Same region as for baseline if not-visible;

Additional/changed findings (e.g. artefacts etc.): ___________________________________

Characteristics : |___|
1 = No residual hyper-intense signal on b800-b1500 images or low/no ADC at former tumor location
2 = Small but obvious hyper-intense signal at former tumor location on b800-b1500 images; ADC may be low, but is not measurable
3 = Hyper-intense signal at former tumor location on b800-b1500 images, combined with measurable low ADC
4= Hyper-intense signal at former tumor location on b800-b1500 images, combined with measurable high ADC
5=Other, specify (see table 1):____________________________

Conclusion:

Response Mamma (visual): |___| *1 = Definitely radiologic complete response*
2 = Probably complete radiologic response
3 = Possible complete radiologic complete
4 = Probable no radiologic complete response
5 = Definitely no radiologic complete response
9 = not evaluable (if so please disclose why)

Remarks:

Table 1. Garcia-Figueiras et al., *Insights into Imaging,* 2019

Table 1 Interpretation of tumor diffusion-weighted images

Signal intensity on high b-value images (b800–b1000)	Relative value on apparent diffusion coefficient (ADC) maps	Signal intensity on T2-weighted images	Interpretation
High	Low	Intermediate	Generally, high cellularity tumor<br>Coagulative necrosis<br>Abscess<br>Rarely high protein content
High	High	High	T2-*shine through* (often proteinaceous fluid)
Low	Low	Low	Fibrous tissue with low water content +/- viable tumor
Low	High	High	Fluid<br>Liquefactive necrosis<br>Lower cellularity/ grade tumor<br>Glandular tissue
Low	High	High	Vasogenic edema (T2-*wash out*)
Low	Low	Variable	Hemorrhagic content (T2-*black out*)
